# Nrf2 activation by the monocarbonyl curcumin derivative GO-Y015 confers cellular protection against arsenite toxicity by reducing intracellular arsenic levels

**DOI:** 10.1038/s41598-026-49334-0

**Published:** 2026-04-18

**Authors:** Md. Tanvir Islam, Hiroki Taguchi, Hiroyuki Yamakoshi, Wang Yinuo, Hiroyuki Shibata, Yoshiharu Iwabuchi, Takashi Toyama, Yoshiro Saito

**Affiliations:** 1 Laboratory of Molecular Biology and Metabolism, Graduate School of Pharmaceutical Sciences, Tohoku University, 6-3 Aoba, Aramaki, Aoba-ku, Sendai, Miyagi 980-8578 Japan; 2 Japan Society for the Promotion of Science (JSPS) Postdoctoral Fellow, Graduate School of Pharmaceutical Sciences, Tohoku University, 6-3 Aoba, Aramaki, Aoba-ku, Sendai, Miyagi 980-8578 Japan; 3 Department of Microbiology, Jashore University of Science and Technology, Jashore, 7408 Bangladesh; 4 Laboratory of Synthetic Chemistry, Graduate School of Pharmaceutical Sciences, Tohoku University, 6-3 Aoba, Aramaki, Aoba-ku, Sendai, Miyagi 980-8578 Japan; 5 Department of Clinical Oncology, Graduate School of Medicine, Akita University, Hondo 1-1-1, Akita, 010-8543 Japan

**Keywords:** Biochemistry, Chemical biology, Drug discovery, Environmental sciences

## Abstract

Curcumin, a bioactive phenolic compound derived from turmeric, attenuates heavy metal toxicity, including inorganic arsenite (As(III)), via activation of the Nrf2–Keap1 signaling pathway; however, its low solubility and stability limit its bioactivity. To address these limitations, we synthesized curcumin derivatives and investigated the cytoprotective effects of a monocarbonyl analog, GO-Y015. GO-Y015 exhibited cytotoxicity comparable to curcumin at concentrations below 8 µM but significantly reduced As(III)-induced cytotoxicity. Pretreatment with GO-Y015 markedly improved cell viability under cytotoxic As(III) exposure, whereas curcumin showed minimal protective effects. Mechanistically, GO-Y015 induced robust Nrf2 activation in a concentration- and time-dependent manner, resulting in increased expression of downstream targets, including HO-1, GCLC, and MRP2. Pharmacological inhibition of Nrf2 (ML385), GSH synthesis (BSO), or MRP transporters (MK571) abolished the cytoprotective effects, indicating dependence on the Nrf2–GSH–MRP axis. Furthermore, ICP-MS analysis demonstrated that GO-Y015 significantly reduced intracellular arsenic accumulation after 2 h exposure. These findings indicate that GO-Y015 enhances arsenic detoxification and reduces cytotoxicity by promoting GSH-dependent conjugation and MRP-mediated efflux.

**Supplementary Information:**

The online version contains supplementary material available at 10.1038/s41598-026-49334-0.

## Introduction

Groundwater is a vital source of drinking water for millions of people worldwide and is also used for cooking, agricultural irrigation and various household purposes. Consequently, arsenic contamination in groundwater is a significant global public health concern. As arsenic is colorless, tasteless and odorless, it is extremely difficult to detect in water, food or air without specialized analysis, which allows chronic exposure to go unnoticed until symptoms appear^1^. The World Health Organization (WHO) has set the permissible limit for arsenic in drinking water at 10 parts per billion (ppb). However, several regions, including Bangladesh, West Bengal, Thailand, Finland, Hungary, Chile, Taiwan, Vietnam, Cambodia, Mexico, Argentina, China and parts of the United States, have reported arsenic levels far exceeding this threshold. Chronic exposure to arsenic has been linked to an increased risk of skin lesions, liver damage, neurological problems, diabetes and skin, lung and bladder cancers^2,3^.

Curcumin is a natural polyphenolic compound derived from the rhizome of Curcuma longa (turmeric). It has been used for centuries in traditional medicine and as a culinary spice in South and Southeast Asia. Extensive in vitro and in vivo studies have demonstrated that curcumin possesses antioxidant, anti-inflammatory, antimicrobial, antiviral, neuroprotective and anti-cancer properties^4,5^, while its bioavailability is low^6^. Curcumin acts as a potent activator of the Nrf2 signaling pathway by covalently modifying the reactive cysteine residues of its repressor protein, Kelch-like ECH-associated protein 1 (Keap1). Once activated, Nrf2 translocate into the nucleus, inducing genes that respond to antioxidant response elements (AREs), e.g. phase II detoxifying enzymes, redox-regulating proteins, and xenobiotic transporters^7,8^. These include heme oxygenase-1 (HO-1), glutamate-cysteine ligase catalytic and modifier subunits (GCLC and GCLM), glutathione S-transferases (GSTs) and multidrug resistance-associated proteins (MRPs). Collectively, these enhance cellular resilience against oxidative and electrophilic stresses^8,9^.

The importance of Nrf2 in heavy metal detoxification has been well established^10^. We previously reported that sulforaphane, a representative Nrf2 activator can reduce toxicity of arsenite (As(III)) and methylmercury^11–14^. In the context of As(III), cellular uptake is facilitated by aquaglyceroporins^15^, after which arsenite undergoes biotransformation to monomethylarsonous acid (MMeAs(III)) or forms arsenic–glutathione conjugates (As(III)–SG) via GST-mediated conjugation^16^. These conjugates are subsequently exported from cells via MRP family transporters, thereby limiting the accumulation of arsenic within cells and reducing its toxicity. Numerous studies have demonstrated that curcumin-mediated activation of Nrf2 increases the expression of these downstream effector genes, thereby improving defense against the cytotoxic effects of harmful metals, including arsenic^5,17,18^.

Despite its promising pharmacological profile and potent capacity to induce Nrf2, curcumin’s clinical potential has been limited by its poor aqueous solubility, low chemical stability under physiological conditions, rapid metabolism and rapid systemic elimination^6^. These restrictions have been addressed through the exploration of various strategies, including the use of nanocarrier formulations, the co-administration of adjuvants, and the structural modification of the curcumin backbone^19,20^. Among these, rational design of curcumin derivatives has received particular attention, and several analogs have demonstrated enhanced bioavailability, improved metabolic stability, and superior pharmacological potency relative to the parent molecule, e.g., monocarbonyl analogs^21^. Structure–activity relationship (SAR) analyses have suggested that making selective modifications to the β-diketone moiety or the phenolic rings could increase the electrophilicity towards the Keap1 cysteine residues, thereby improving the efficiency with which Nrf2 is activated^22^. The beta-diketone moiety in curcumin contributes to its instability, whereas monocarbonyl modification enhances chemical stability^23^. For example, pyrolysis-derived “dieneone curcumin” (deketomin; also known as GO-Y022) exhibited stronger bioactivity than curcumin^24^. Such structure–activity relationship understanding will support the development of novel Nrf2 activator.

In recent efforts, we synthesized a set of structurally modified curcumin derivatives and identified several candidates that exhibit stronger Nrf2-inducing activity and additional anti-diabetic effects than curcumin^25^. GO-Y015, a monocarbonyl derivative of curcumin, emerged among these derivatives as a promising Nrf2 activator compound. In this study, we evaluated cytoprotective effects of GO-Y015 against arsenite-induced toxicity, investigating whether its protective action involves enhanced Nrf2–Keap1 axis activation. We hypothesized that GO-Y015 would be more effective than curcumin at mitigating arsenite toxicity due to its improved chemical stability and bioactivity. It has previously been established that Nrf2 activation reduces the toxicity of arsenic in HepG2 cells as well as primary mouse hepatocytes; therefore, we focused on liver cells—which play a key role in arsenic metabolism—and used HepG2 cells as a limited model here^14,26^.

## Material and method

### Materials

Curcumin was purchased from Wako Pure Chemical Industries, Ltd., Osaka, Japan (038-04921). Curcumin derivative, GO-Y015 was synthesized as described previously^25^. Curcumin and GO-Y015 were dissolved in DMSO and divided into smaller aliquots and frozen before use. AsIII was purchased from Toronto Research Chemicals, Canada (S080868); MK571 (L-660711) (S0505), L-Buthionine sulfoximine (BSO) (S9728L), and ML385 (S8790) were purchased from Selleck, Japan; All reagents used in this study were of the highest purity and suitable for research-grade analysis. FBS used in this study (Sigma, SA, USA. Lot.BCCC5944) containing approximately 8 ppb of selenium, which affect arsenite toxicity and experimental reproducibility^27,28^.

### Cell culture

The HepG2 cell line (derived from human liver carcinoma, JCRB cell bank, Osaka, Japan) was used for all cell culture experiments in this study. HepG2 cells were cultured in Dulbecco’s Modified Eagle Medium (4.5 g/L Glucose) with L-Gln and Sodium Pyruvate with 10% fetal bovine serum, 100 U/mL and 100 μg/mL penicillin–streptomycin in a humidified incubator at 37 °C, 5% CO_2_, and 95% ambient air. For cell maintenance, HepG2 cells were cultured in a 10 cm dish passaged at a cell density of 10% or 20% and cultured to an 80% sub confluent state. In the present study, HepG2 was seeded 24 h before the experiments.

### Cell viability

HepG2 cells were seeded at a density of 1 × 10^4^ cells per well in a 96-well plate and cultured for 24 h. For evaluating cell viability by AsIII, curcumin and GO-Y015, different concentration of them were added to achieve a final volume of 100 μL per well. After 24 h of treatment, media were then replaced with media containing 10% alamarBlue reagent (Thermo Fisher, OR, USA) and incubated for 3 h. The absorbance at 570 nm was then measured using a plate reader (Molecular Devices, SpectraMax iD5, CA, USA).

### Western blotting

Cells were lysed using a 2% SDS buffer [0.05 M Tris–HCL (pH6.8), 10% Glycerol, 2% SDS] to extract proteins and boiled for 95 °C, 10 min for analysis. The DC protein assay kit (Bio-Rad, CA, USA) was used for quantifying protein’s concentration with bovine serum albumin, F-V, pH5.2 (Nacalai Tesque, Kyoto, Japan) as the standard. Protein samples were separated by sodium dodecyl sulfate–polyacrylamide gel electrophoresis (SDS-PAGE) and transferred onto a polyvinylidene difluoride (PVDF) membrane using the Criterion Blotter (Bio-Rad, CA, USA). The membrane was blocked with 5% skim milk. The membrane was rinsed with TBST and, protein immunoblotting was performed using the primary antibodies anti-Nrf2 [sc365949] and anti-HO-1 [sc390991] from Santa Cruz Biotechnology, TX, USA; anti-GAPDH [015-25473] was from Wako Pure Chemical (Osaka, Japan). Primary antibodies were incubated overnight at 4 °C, and secondary antibody combined with HRP were incubated for 1 h at room temperature with gentle shaking. Chemiluminoscence was detected using an ImmunoStar LD and ZETA kit (Wako Pure Chemical).

### Reverse transcription-quantitative PCR (RT-qPCR)

Total RNA was extracted using ISOGEN II (Nippon Gene, Tokyo, Japan), according to manufactures instruction. RNA concentration was determined with a NanoDrop ND-1000 spectrophotometer (Thermo Fisher Scientific, Warrington, UK). Complementary DNA (cDNA) was synthesized using the PrimeScript RT reagent kit (Takara Bio, Shiga, Japan) according to the manufacturer’s protocol. Primers targeting the genes of interest were diluted to a final concentration of 10 μM in nuclease-free water (Qiagen, Hilden, Germany). The cDNA samples were diluted 1:10 and used as templates. For RT-qPCR, each 20 μL reaction contained 4 μL of template cDNA, 5 μL of Power SYBR Green PCR Master Mix (Thermo Fisher Scientific), and 1 μL each of forward and reverse primers (sequences are follows). Human GAPDH; F-GCACCGTCAAGGCTGAGAAC, R- TGGTGAAGACGCCAGTGGA. MRP2; F-CCCTGCTGTTCGATATACCAAT, R- TCGAGAGAATCCAGAATAGGGA. GCLC; F-GGAGACCAGAGTATGGGAGTT, R- CCGGCGTTTTCGCATGTTG. Primers were synthesized by Fasmac (Kanagawa, Japan). RT-qPCR was carried out using the Thermal Cycler Dice Real-Time System (Takara) under standard cycling conditions. GAPDH was used as the internal control gene.

### Measurement of intracellular GSH level by high-performance liquid chromatography coupled with an electrochemical detector (HPLC-ECD)

The cellular glutathione (GSH) content was determined using high-performance liquid chromatography (HPLC)-electrochemical detector (ECD) analysis, which was slightly modified from our previous method^12^. Briefly, cells were harvested with 1 mM EDTA and sonicated to rupture the cells. After centrifugation, the supernatant was subjected to an HTEC-500 HPLC-ECD system (EiCOM, Kyoto, Japan) equipped with an InertSustain C18 column (150 mm × 2.1 mm, i.d.; GL Science, Tokyo, Japan). A mobile phase consisting of 20 mM ammonium phosphate buffer (pH 2.5) and 1.8% acetonitrile was used to separate GSH. The flow rate was fixed at 0.25 mL/min, and the retention time of GSH was 3.8 min under these conditions. The electrode potential was 300 V, and the time constant was 1.5 s. The GSH standard curve was prepared using 077-02011 (Wako Pure Chemical, Osaka, Japan). The sample was measured at a dilution ratio that was linear within the calibration curve. The protein concentration was measured using a DC protein assay kit and was used for normalization.

### Determination of Arsenic concentration by ICP-MS

Samples were acid-digested prior to elemental analysis. Briefly, each sample was mixed with 250 μL of concentrated nitric acid (70%) and decomposed using a microwave digestion system (ETHOS EASY, Milestone General, Kanagawa, Japan). Digestion was carried out in sealed high-pressure quartz vessels at 160 °C with a maximum pressure of 80 bar for 30 min. Following digestion and cooling to ambient temperature, the resulting solutions were diluted with ultrapure water to a total volume of 1.0 mL (final nitric acid concentration: 17.5%) and subjected to ICP-MS analysis. Arsenic analysis was performed using an Agilent 8900 inductively coupled plasma mass spectrometer (Agilent Technologies, Santa Clara, CA, USA). Both ^75^As and its oxygen reaction product, ^75^As^16^O^+^ (m/z 91), were monitored for quantification. Measurements were conducted in oxygen reaction mode to promote the formation of arsenic oxide ions, thereby minimizing argon-based polyatomic interferences. A mixed internal standard solution containing Be, Y, In, Te, and Bi was prepared in 17.5% nitric acid and continuously introduced online with the samples. Arsenic concentrations were quantified using external calibration with internal standard correction. Calibration standards were prepared from a certified arsenic standard solution (Kanto Chemical Co., Inc., Tokyo, Japan).

### Statistics and reproducibility

**Fig. 1:**
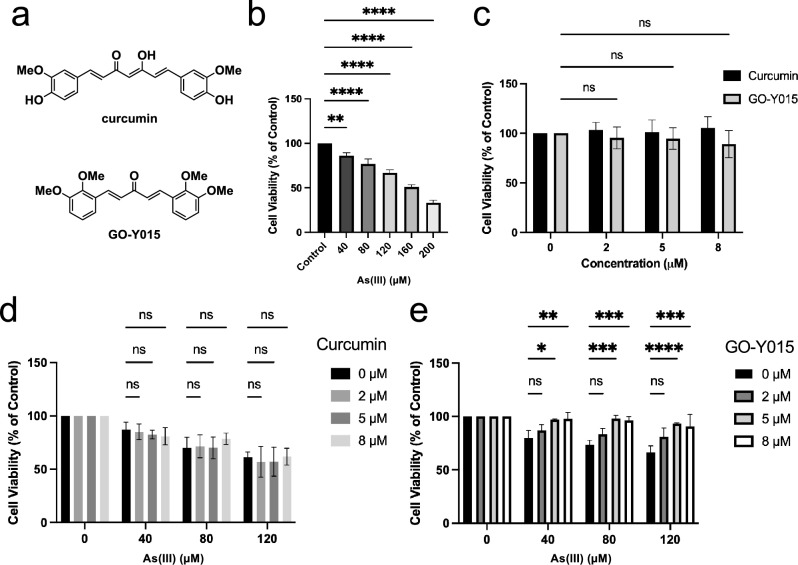
Effect of curcumin and GO-Y015 against As(III) toxicity in HepG2 cell. (**a**) Chemical structure of curcumin and GO-Y015. (**b**) Cytotoxicity of As(III) exposure was evaluated using the Alamar Blue assay. HepG2 cells were seeded in a 96-well plate at a density of 0.5 × 10^4^ cells/well in 100 µL medium and cultured in High Glucose DMEM for 24 h. As(III) was added to achieve 0–200 µM. After 24 h, alamarBlue assay was performed. n = 3, Mean ± S.D., **P < 0.01, ****P < 0.0001 vs. DMSO, One-way ANOVA with Dunnett’s post hoc test.. (**c**) Cytotoxicity of curcumin and GO-Y015 were evaluated using the Alamar Blue assay. Curcumin and GO-Y015 were added to achieve 0–8 µM. After 6 h, alamarBlue assay was performed. n = 3, Mean ± S.D., **P < 0.01, ****P < 0.0001 vs. DMSO, One-way ANOVA with Dunnett’s post hoc test. (**d**) HepG2 cells were pre-treated with Curcumin at 2–8 µM. After 6 h, media were changed with fresh High Glucose DMEM containing As(III) at 0–120 µM. After 16 h, alamarBlue assay was performed. n = 3, Mean ± S.D., **P < 0.01, ****P < 0.0001 vs. DMSO, One-way ANOVA with Dunnett’s post hoc test. (**e**) HepG2 cells were pre-treated with GO-Y015 at 2–8 µM. After 6 h, media were changed with fresh High Glucose DMEM containing As(III) at 0–120 µM. After 16 h, alamarBlue assay was performed. n = 3, Mean ± S.D., **P < 0.01, ****P < 0.0001 vs. DMSO, One-way ANOVA with Dunnett’s post hoc test.

**Fig. 2:**
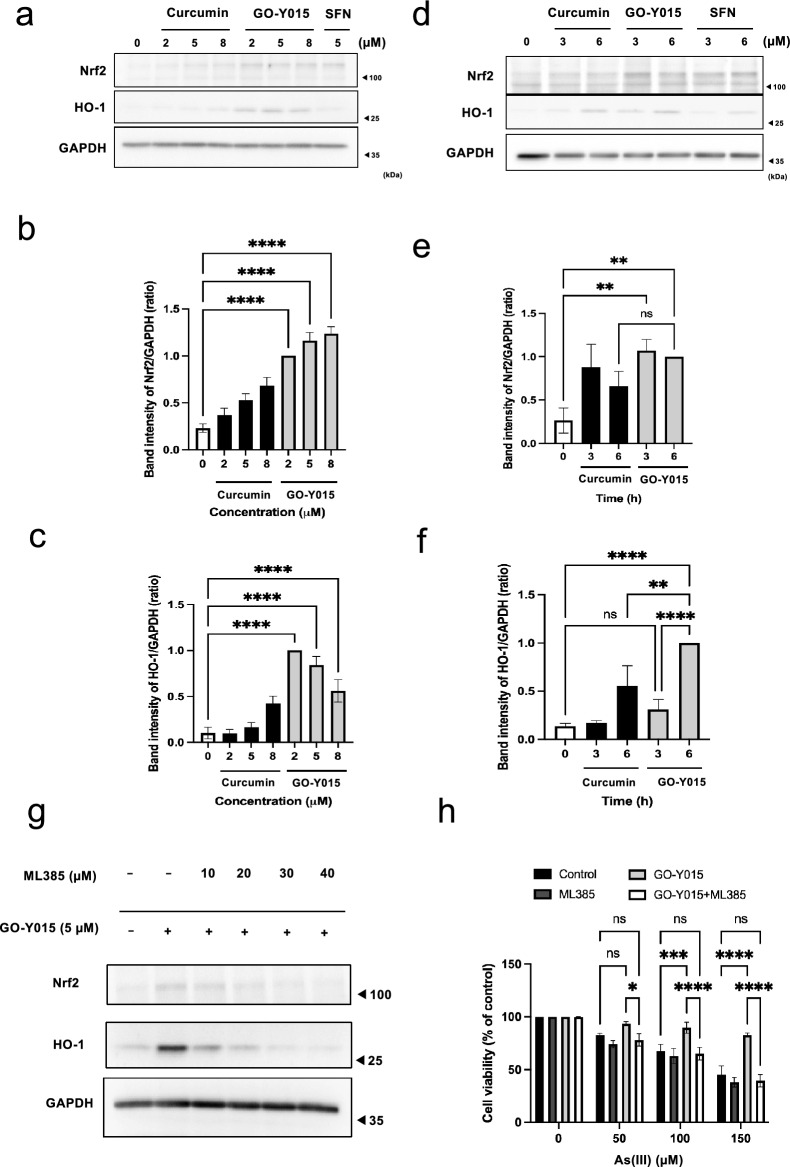
Nrf2 activation by curcumin and GO-Y015. (**a**–**c**) HepG2 cells were seeded in a 12-well plate at a density of 1 × 10^5^ cells/well in 1,000 µL medium and cultured in High Glucose DMEM for 24 h. For concentration-course experiments, curcumin (2–8 µM), GO-Y015 (2–8 µM) sulforaphane (5 µM) were added. After 6 h, cells were harvested using 2% SDS buffer for protein and analyzed by Western blot (n = 3). Data were shown as mean ± S.D., *P < 0.05, **P < 0.01, ***P < 0.001, ****P < 0.0001 vs. DMSO, One-way ANOVA with Dunnett’s post hoc test. (**d**–**f**) For time-course experiments, curcumin (5 µM), GO-Y015 (5 µM) sulforaphane (5 µM), or DMSO were added at 0 h. Samples were collected after 3 and 6 h of incubation. Then the cells were harvested using 2% SDS buffer for protein and analyzed by Western blot (n = 3). mean ± S.D., *P < 0.05, **P < 0.01, ***P < 0.001, ****P < 0.0001 vs. DMSO, One-way ANOVA with Dunnett’s post hoc test. (**g**) The cells were co-treated with GO-Y015 (5 or 8 µM) and ML385 (0–40 µM). After 6 h, the cells were harvested using 2% SDS buffer for protein and analyzed by Western blot (n = 3). (**h**) HepG2 cells were pre-treated with GO-Y015 (5 µM), ML385 (40 µM) and GO-Y015 (5 µM) + ML385 (40 µM). After 6 h, media were changed with fresh High Glucose DMEM containing As(III) at 0–150 µM. After 16 h, alamarBlue assay was performed. n = 3, Mean ± S.D., **P < 0.01, ****P < 0.0001 vs. DMSO, One-way ANOVA with Dunnett’s post hoc test.

**Fig. 3:**
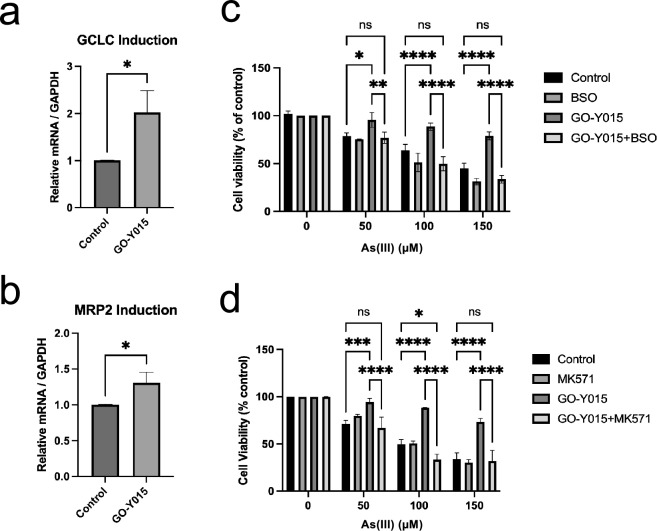
Role of GCL and MRP in chemoprevention of As(III) toxicity by GO-Y015. (**a**, **b**) HepG2 cells were seeded in a 12-well plate at a density of 1 × 10^5^ cells/well in 1,000 µL medium and cultured in High Glucose DMEM for 24 h. After 24 h, GO-Y015 (5 µM) was added and incubated for 6 h. After that cells were harvested using ISOGEN II (for RNA) and analyzed by RT-qPCR (n = 3). Data were shown as mean ± S.D., *P < 0.05, **P < 0.01, ***P < 0.001, ****P < 0.0001 vs. DMSO, One-way ANOVA with Dunnett’s post hoc test. (**c**) HepG2 cells were pre-treated with GO-Y015 (5 µM), BSO (1 µM) and GO-Y015 (5 µM) + BSO (1 µM). After 6 h, media were changed with fresh High Glucose DMEM containing As(III) at 0–150 µM. After 16 h, alamarBlue assay was performed. n = 3, Mean ± S.D., **P < 0.01, ****P < 0.0001 vs. DMSO, One-way ANOVA with Dunnett’s post hoc test. (**d**) HepG2 cells were pre-treated with GO-Y015 (5 µM), MK571 (50 µM) and GO-Y015 (5 µM) + MK571 (50 µM). After 6 h, media were changed with fresh High Glucose DMEM containing As(III) at 0–150 µM. After 16 h, alamarBlue assay was performed. n = 3, Mean ± S.D., **P < 0.01, ****P < 0.0001 vs. DMSO, One-way ANOVA with Dunnett’s post hoc test.

**Fig. 4:**
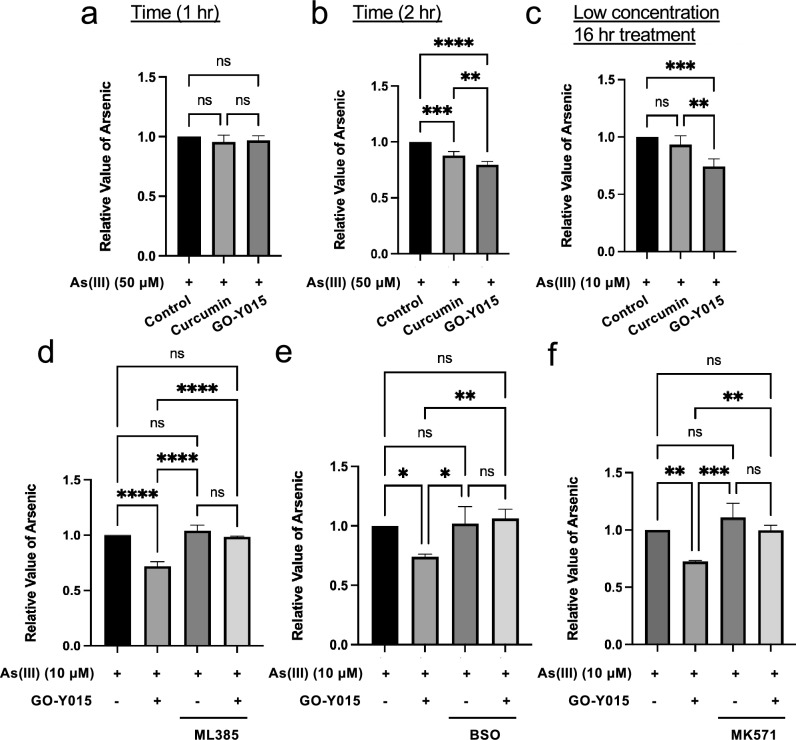
Effect of GO-Y015 on the accumulation of intracellular arsenic in HepG2 cells. (**a**–**c**) HepG2 cells were seeded in a 12-well plate at a density of 2.5 × 10^5^ cells/well in 1,000 µL medium and cultured in High Glucose DMEM for 24 h. Media were changed and cells were pre-treated with curcumin (5 µM) or GO-Y015 (5 µM). After 6 h, media were changed with fresh High Glucose DMEM containing As(III) (50 µM). After 1 h (**a**) or 2 h (**b**), intracellular arsenic level was determined by ICP/MS. (**c**) The cells were treated with As(III) (10 µM) as same condition as above and after 16 h, intracellular arsenic level was determined. The protein concentration was used for the normalization (n = 3). Data were shown as mean ± S.D.*P < 0.05, **P < 0.01, ***P < 0.001, ****P < 0.0001 (compared to control, One-way ANOVA + Dunnett’s test). (**d**–**f**) The cells were treated with, (**d**) GO-Y015 (5 µM), ML385 (5 µM) and GO-Y015 (5 µM) + ML385 (5 µM); or (**e**) GO-Y015 (5 µM), BSO (10 µM) and GO-Y015 (5 µM) + BSO (10 µM); or (**f**) GO-Y015 (5 µM), MK571 (20 µM) and GO-Y015 (5 µM) + MK571 (20 µM). After 6 h, media were changed with fresh High Glucose DMEM containing As(III) (10 µM). After 16 h, intracellular arsenic level was determined by ICP/MS. The protein concentration was used for the normalization (n = 3). Data were shown as mean ± S.D.*P < 0.05, **P < 0.01, ***P < 0.001, ****P < 0.0001 (compared to control, One-way ANOVA + Dunnett’s test).

**Fig. 5:**
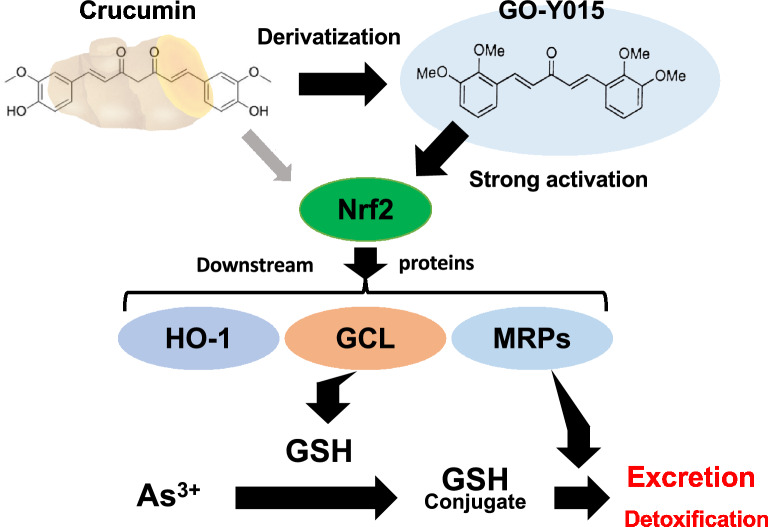
Overview of the study; GO-Y015 protects As(III) toxicity through Nrf2 activation.

For statistical processing, Student’s t-test was used for comparison between two groups. A multiple comparison test (one-way ANOVA, two-way ANOVA, post hoc test Dunnett’s method or Tukey’s method) was used to detect significant differences between three or more groups by Prism10 (ver10.2.2). EC50 was alsso evaluated by using Prism 10. The difference was significant when the probability value was 5% or less. The data are expressed as mean ± standard deviation (S.D.). Three replicates (biological replicates) were obtained with independent experiments. Band intensity was quantified by ImageJ 1.53i (National Institutes of Health, USA). Detailed sample sizes and statistical methods are described in the figure legends (Figs. 1, 2, 3, 4, 5).

## Results

### Protective effect of curcumin and monocarbonyl GO-Y0015 on the arsenite-induced cytotoxicity in HepG2 cells

Monocarbonyl curcumin derivative GO-Y015 is a more effective Nrf2 activator than curcumin^25^ and is expected to be an effective chemopreventive agent that reduces arsenite (As(III)) toxicity (Fig. 1a). We investigated the protective effects of curcuminoids against arsenite (As(III)) toxicity by determining the cytotoxic dose of arsenite in HepG2 cells, as curcumin has been reported to activate Nrf2 in the liver. Exposure to As(III) for 24 h resulted in a dose-dependent reduction in HepG2 cell viability, with a significant cytotoxic effect at concentrations greater than 40 µM (Fig. 1b). Next, we evaluated sub-cytotoxic doses of curcuminoids (curcumin and GO-Y015). These curcuminoids exhibited no toxicity at concentrations below 8 µM within 24 h (Fig. 1c).

Previous reports have suggested that pretreating cells with curcumin before adding toxicants is an effective way to evaluate its cytoprotective effects^29^. We pretreated HepG2 cells with a sub-cytotoxic dose of curcuminoids for six hours, then treated the cells with a cytotoxic dose of As(III) for the next 16 h. The results indicated that curcumin does not protect against As(III) toxicity under these conditions. Toxic does 50 (TD_50_) of As(III) was 165.6 µM and curcumin treatment failed to affect this TD_50_ value. However, GO-Y015 effectively protected against As(III) toxicity when pretreated at concentrations greater than 5 µM (Figs. 1d,e), and TD50 of As(III) was increased to 261.5 µM (estimated from Fig. 3d). In conclusion, our results suggest that GO-Y015 is a more effective chemopreventive agent than curcumin against As(III).

### GO-Y015 protects As(III) toxicity through Nrf2 activation

Our previous study indicated Nrf2 activator, sulforaphane (SFN), protects As(III) and methylmercury toxicity Nrf2 dependently^12,14^. Therefore, we hypothesized that GO-Y015 would suppress As(III) toxicity by activating Nrf2 as well. At the steady state, Nrf2 undergoes breakdown via the ubiquitin–proteasome pathway, which is mediated by Keap1, a Nrf2 suppressor. Once electrophiles, such as SFN and curcumin, bind to the Cys residues of Keap1, Nrf2 accumulates and is translocated to the nucleus, where it drives the expression of downstream genes, such as heme oxygenase-1 (HO-1). Thus, the accumulation of Nrf2 and the induction of HO-1 can be hallmarks of Nrf2 activation^8^.

Treating cells with 2–8 µM curcumin or GO-Y015 for six hours under the conditions shown in Fig. 1 resulted in Nrf2 accumulation and HO-1 protein expression increases in a dose-dependent manner. The effect of GO-Y015 was stronger than that of curcumin (Fig. 2A–C). The time-dependent accumulation of Nrf2 and HO-1 induction was also higher with GO-Y015 (Figs. 2d–f). These results indicate that GO-Y015 is a stronger Nrf2 activator than curcumin. Next, we used ML385^30^, which is an inhibitor of Nrf2 to evaluate the involvement of Nrf2 in cytoprotective effect of GO-Y015. First, we confirmed that ML385 was nontoxic to HepG2 cells at exposures of 10, 20, 30, and 40 µM. Next, we treated HepG2 cells with 5 µM GO-Y015 and ML385 concomitantly for 6 h. We observed that, as the concentration of ML385 increased, the level of Nrf2 and HO-1 proteins gradually decreased in a dose-dependent manner and was completely inhibited at 40 µM (Fig. 2g). Under these conditions, the co-exposure of ML385 and GO-Y015 completely abolished the cytoprotective effect of GO-Y015 against As(III)-induced cytotoxicity (Fig. 2h). These results suggest that the chemopreventive effect of GO-Y015 against As(III) depends on Nrf2 activation.

### Mechanism underlying cytoprotective effect of GO-Y015 against As(III) via Nrf2

Previous reports indicated SFN protect As(III) toxicity through induction of glutamate-cysteine ligase (GCL) and multidrug resistant protein (MRPs) via Nrf2, because As(III) undergo glutathione (GSH) conjugation and excreted to extracellular space through MRP for excretion^11,14^.

Here we evaluated the induction of GCLC, a catalytic subunit of GCL, and MRP2, which is responsible for the excretion of GSH-conjugate in liver. The results indicated induction of both genes by GO-Y015 rather than curcumin (Fig. 3a,b). The intracellular GSH level was measured using high-performance liquid chromatography coupled with an electrochemical detector (HPLC-ECD), which revealed that GO-Y015 increased it by approximately threefold (see Supplementary Fig. 1). Buthionine sulfoximine (BSO), an inhibitor for GCL, or MK571, an inhibitor for MRPs, canceled the protective effect of GO-Y015 against As(III) toxicity (Fig. 3c,d d).

The GCL-produced GSH and MRP excretion pathway could decrease intracellular As(III) accumulation. We measured the total As level in cells using ICP-MS. First, we pretreated HepG2 cells with 5 µM curcumin and GO-Y015 for 6 h. Then, we added 50 µM (toxic level) and 10 µM (subtoxic level) of As(III) to the cells. According to our observations in Fig. 1, 50 µM As(III) exposure was toxic to HepG2 cells after 16 h of treatment. Therefore, we exposed curcumin- and GO-Y015-treated HepG2 cells for shorter periods (1 and 2 h). The results showed no significant difference in the intracellular arsenic (As) level between curcumin- and GO-Y015-treated cells after one hour of exposure. However, after two hours, the intracellular As level was significantly lower in GO-Y015-treated cells compared with the control group and curcumin-treated cells (Fig. 4a,b). Similarly, we found a significantly lower level of As in GO-Y015-treated cells compared to the control group and curcumin-treated cells after sub-toxic exposure to As(III) (Fig. 4c). This indicates that non-specific As accumulation accompanied by cellular damage and its protection by GO-Y015 is not involved. These results suggest that GO-Y015 enhances As excretion, thereby lowering its accumulation in cells. This process may be involved in Nrf2-mediated cytoprotection against As(III).

Next, we measured intracellular As levels directly in Nrf2-, GSH-, and MRP-inhibited HepG2 cells. In all conditions, intracellular As levels increased in cells co-treated with As(III) and the inhibitors compared to control cells (Fig. 4d–f). However, the increase in intracellular As levels observed in cells treated with As(III) and different inhibitors was unaffected by pre-treatment with GO-Y015 (Fig. 4d–f). These results demonstrate that As(III) is metabolized into an arsenite-glutathione adduct (As(III)-SG), which is catalyzed by glutathione S-transferases (GSTs) and excreted from cells into the extracellular space through multidrug resistance proteins (MRPs) in a Nrf2-dependent manner (Fig. 5).

## Discussion

Curcumin, sulforaphane, isothiocyanates, epigallocatechin gallate, 6-gingerol, and resveratrol are representative phytochemicals known to induce Nrf2 and reduce arsenic-mediated cellular injury^31^. However, their modest electrophilicity, low abundance in plant matrices, poor metabolic stability, and limited bioavailability restrict their translational potential^6^. Therefore, structural modifications, analog design, nanocarrier delivery, and combination electrophile formulations have been explored to improve cellular potency and pharmacokinetic properties^32^. In this context, several synthetic curcuminoid derivatives have been generated that exhibit reduced intrinsic toxicity while retaining or enhancing their Nrf2-activating capacity compared to curcumin. GO-Y015, a structurally engineered curcuminoid, has been reported to induce Nrf2 more effectively than curcumin and other parent compounds. In the present study, we evaluated GO-Y015’s ability to attenuate inorganic arsenite (As(III))-induced cytotoxicity in HepG2 cells.

Curcumin itself is not an effective activator of Nrf2. To overcome this limitation, the stability of synthetic curcumin derivatives has been improved. The presence of a 1,3-dicarbonyl group, for example, contributes to curcumin’s instability. GO-Y015, a monocarbonyl derivative of curcumin, has a modified β-diketone moiety to enhance chemical stability. Previous studies have suggested that monocarbonyl curcumin analogues exhibit enhanced stability under physiological conditions compared to curcumin, which undergoes rapid degradation and metabolism. Therefore, GO-Y015 is expected to have improved chemical stability and better pharmacological properties than curcumin^33,34^.

We found that pretreatment with GO-Y015 significantly increased cell survival following exposure to As(III), whereas curcumin had no notable cytoprotective effect under similar conditions. GO-Y015 increased Nrf2 accumulation and elevated the expression of downstream target genes, including GCLC and MRP2. GCLC is involved in GSH synthesis, and MRP2 is involved in GSH-conjugate export. Inhibiting Nrf2 (with ML385), GSH synthesis (with BSO), or MRP-mediated export (with MK-571) abolished GO-Y015’s cytoprotective effect and increased intracellular arsenic accumulation. This demonstrates that GO-Y015-mediated Nrf2 activation primarily exerts its protective effect through enhanced GSH conjugation and arsenic efflux.

These findings align with previous reports showing that sulforaphane, isothiocyanates, and other Nrf2 activators reduce arsenic accumulation in hepatocyte-derived cell lines. This occurs by promoting the formation of arsenic-GSH conjugates, which are then exported via MRP family transporters^14^. Because As(III) is predominantly detoxified through GSH-dependent conjugation, the increased flux through the GSH-MRP axis caused by GO-Y015 provides a plausible explanation, from a mechanistic standpoint, for the decrease in intracellular arsenic levels and cytotoxicity that was observed. Our results reinforce the idea that arsenic burden, rather than oxidative stress, is a key determinant of toxicity. They also suggest that the induction of arsenic export machinery is an efficient cellular defense mechanism.

Several studies have reported that chronic arsenic exposure depletes glutathione (GSH) levels and disturbs redox homeostasis, making cells more susceptible to oxidative injury. Therefore, the Nrf2–GSH–MRP pathway represents an important compensatory module for maintaining intracellular redox balance under arsenic stress. Demonstrating that GO-Y015 activates this axis more effectively than curcumin suggests that modifying the structure of phytochemical electrophiles can create analogs with enhanced chemopreventive properties^35^. These findings may be relevant to environmental toxicology because electrophilic stressors from pollutants often affect Nrf2-modulated cellular defense systems.

While Nrf2 activation is widely recognized for its cytoprotective and anti-inflammatory effects, sustained Nrf2 upregulation has been linked to increased tumor survival and chemoresistance in certain contexts^7^. Although our study focused on an acute toxicity model, the potential dual roles of Nrf2 must be considered for translational applications. A significant advantage of phytochemical-type electrophiles, such as curcumin analogues, is their reversible, cysteine-dependent activation mechanism, which generally prevents the irreversible modification of Keap1.

Nonetheless, long-term in vivo studies are needed to characterize the safety of chronic GO-Y015 exposure, particularly in cancer-prone tissues. Another important limitation of our study is the lack of pharmacokinetic parameters, including aqueous solubility, metabolic stability, and systemic clearance, and in vivo validation. The metabolism, distribution, and biotransformation of electrophilic phytochemicals differ substantially between in vitro and in vivo systems due to hepatic metabolism, first-pass clearance, plasma protein binding, and conjugation pathways. In previous reports, GO-Y022 has weak potential to activate Nrf2, which indicate that methoxy gropus which contributable to hydrophobicity is involved in efficient Nrf2 activation^25^.

Curcumin itself suffers from poor bioavailability, rapid glucuronidation and sulfation, and limited stability in plasma^6^. GO-Y015 has been shown to have superior Nrf2-inducing activity in HepG2 cells; however, its pharmacokinetic profile remains uncharacterized. To determine whether GO-Y015 can reach physiologically relevant concentrations in target organs, parameters such as oral absorption, metabolic stability, half-life, tissue distribution (particularly hepatic accumulation), and excretion routes would need to be assessed. Additionally, our model used acute exposure to arsenite (As(III)) at cytotoxic concentrations. However, environmental exposure to arsenic typically occurs chronically at lower doses through contaminated water and food. This chronic exposure results in progressive metabolic, endocrine, and inflammatory dysregulation rather than overt cell death. Therefore, it is important to evaluate whether GO-Y015 mitigates mitochondrial dysfunction, DNA damage, epigenetic modifications, and chronic oxidative stress under prolonged, low-dose As(III) exposure. Furthermore, arsenic speciation varies widely across human exposure scenarios, and the response to trivalent inorganic arsenic may differ substantially from the response to pentavalent (As(V)) metabolites or methylated intermediates, which are processed by distinct detoxification pathways^16^. Recently, we discovered that selenium metabolism suppresses arsenic toxicity from the ferroptosis perspective^27^. However, since various electrophilic substances, such as SFN and GO-Y015^25,36^, inhibit selenium metabolism, the mechanisms by which cell death is suppressed may differ.

Despite these limitations, our findings contribute to the growing body of evidence suggesting that structurally optimized phytochemical electrophiles are a viable strategy for mitigating metal and metalloid toxicity. Since arsenic contamination disproportionately affects low-resource settings, safe, inexpensive, orally bioavailable chemopreventive agents would offer meaningful public health benefits if proven effective in chronic exposure models. Therefore, future studies should investigate the pharmacokinetics, oral bioavailability, metabolic fate, and long-term safety of GO-Y015 in appropriate in vivo arsenic exposure models. Additionally, formulation strategies such as nanoparticle encapsulation, lipid carriers, or prodrug approaches may enhance the stability and delivery efficiency of curcumin-derived compounds.

In conclusion, we demonstrate that the curcumin derivative GO-Y015 potently induces Nrf2 and enhances GSH-dependent arsenite (As(III)) detoxification and efflux. This leads to decreased intracellular arsenite accumulation and reduced cytotoxicity in HepG2 cells. These results suggest that structurally engineered phytochemical electrophiles have the potential to be used as chemopreventive agents for As(III)-induced toxicity. However, comprehensive in vivo validation is necessary to determine their feasibility and safety.

## Supplementary Information

Supplementary Information.

## Data Availability

The datasets used and/or analyzed during the current study available from the corresponding author on reasonable request. The uncropped gels/blots data were shown in the supplemental Figures 2–4.
