# Supplementary material for: Nrf2 activation by the monocarbonyl curcumin derivative GO-Y015 confers cellular protection against arsenite toxicity by reducing intracellular arsenic levels
Source: Sci Rep. 2026 Apr 18;16:18048. doi: 10.1038/s41598-026-49334-0 (PMC13254365; doi:10.1038/s41598-026-49334-0)
Supplement: Supplementary file 1 — Supplementary Information. [file 41598_2026_49334_MOESM1_ESM.pdf]

Supplemental figure1 (uncropped figure 2a)

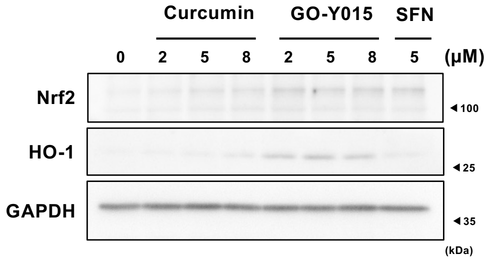

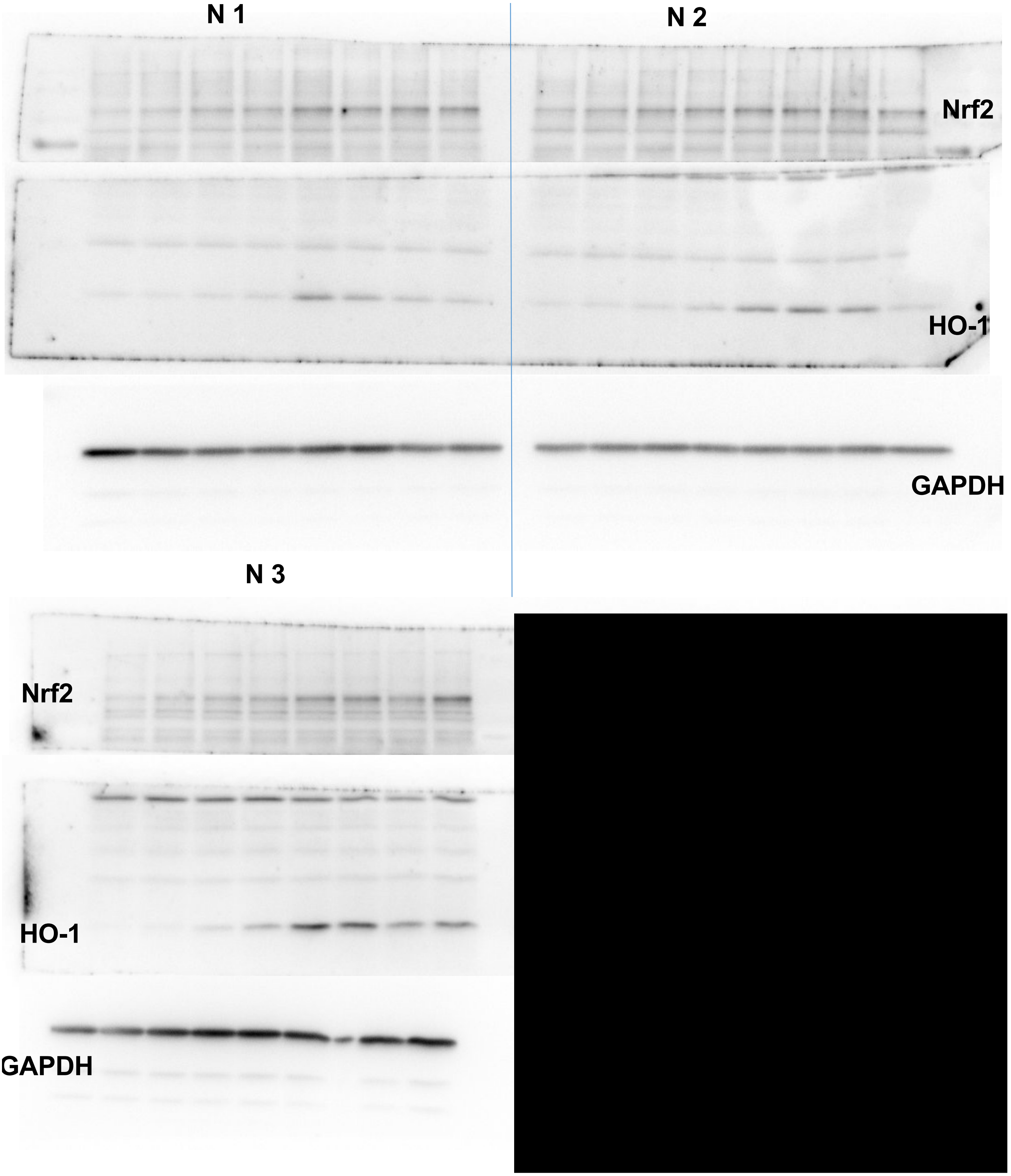

Supplemental figure2 (uncropped figure 2d)

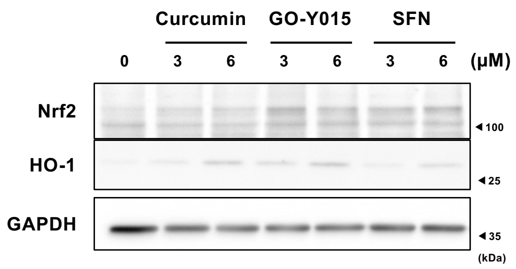

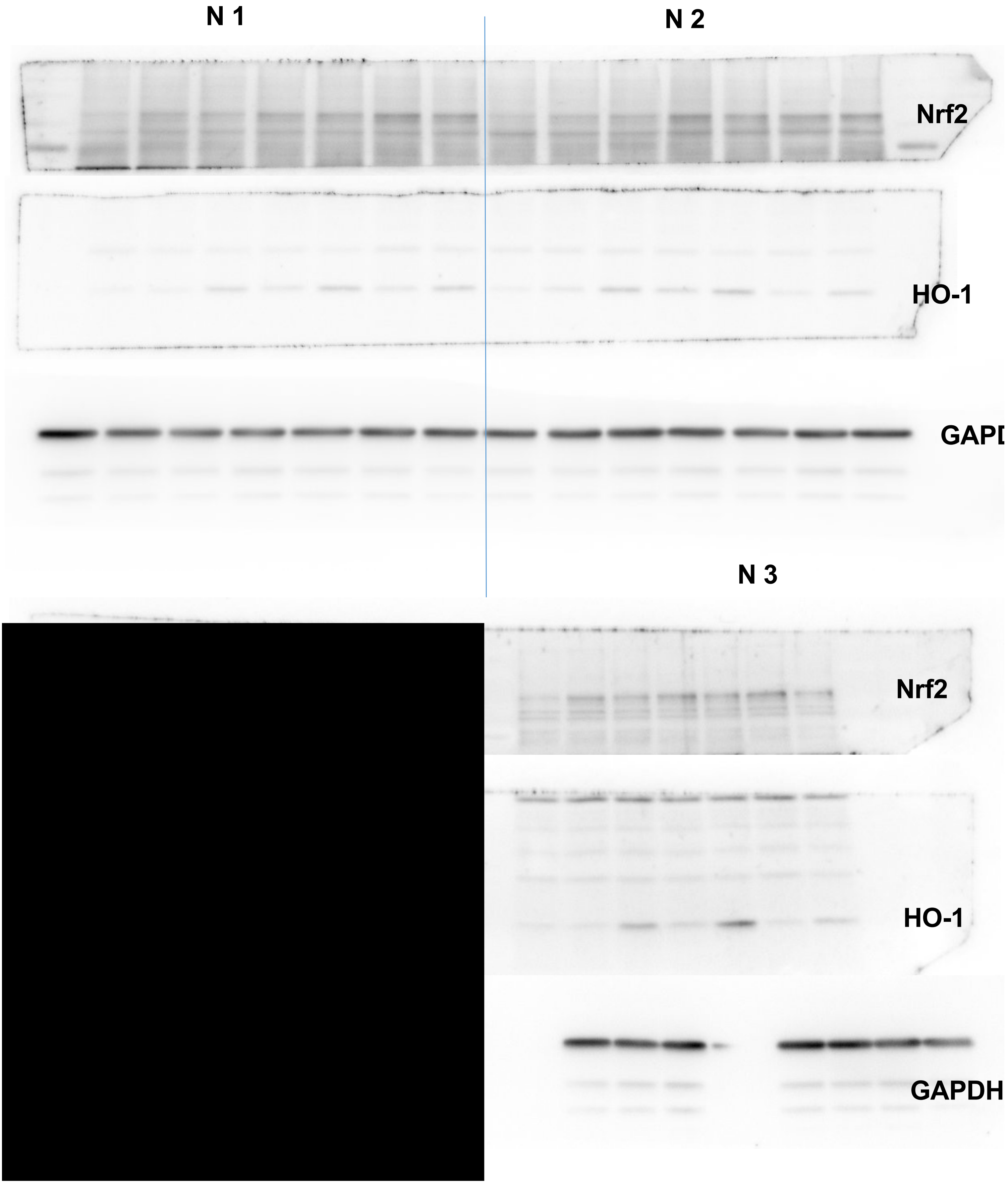

Supplemental figure3 (uncropped figure 2g)

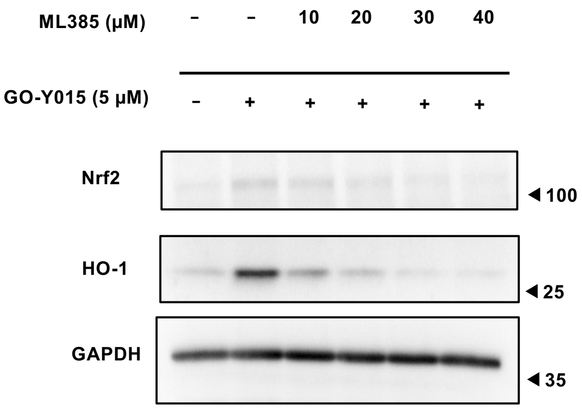

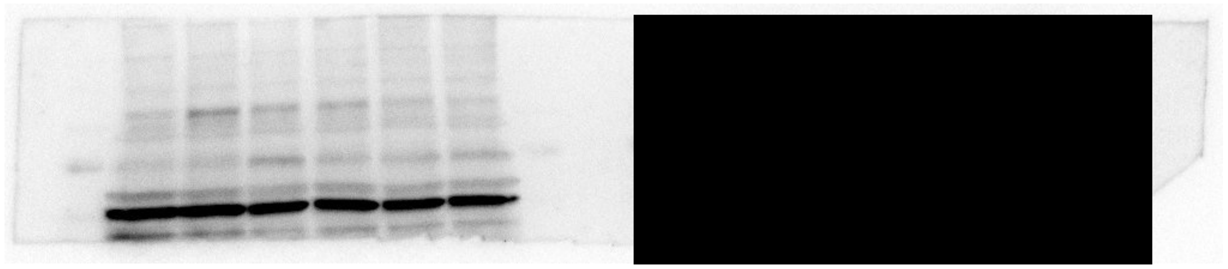

Nrf2

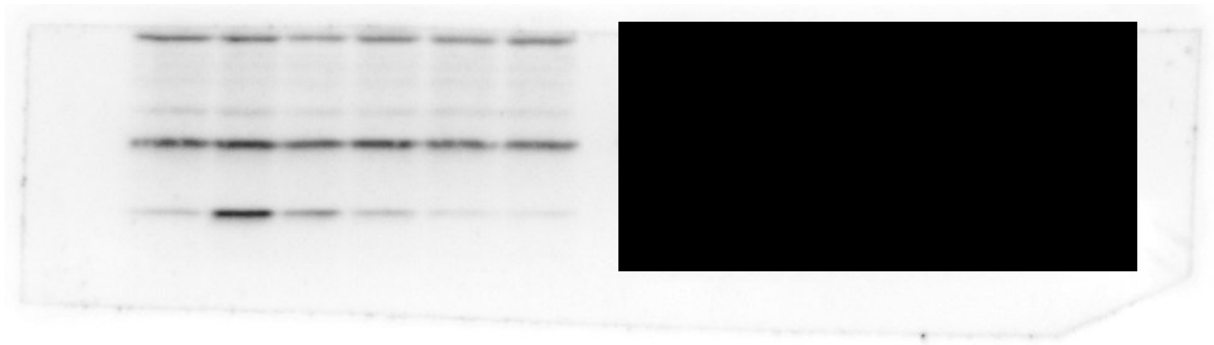

HO-1

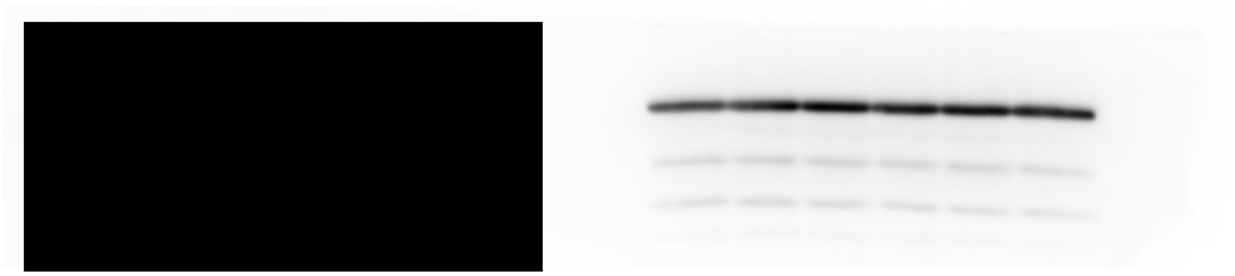

GAPDH
